# Influenza A Virus Encoding Secreted Gaussia Luciferase as Useful Tool to Analyze Viral Replication and Its Inhibition by Antiviral Compounds and Cellular Proteins

**DOI:** 10.1371/journal.pone.0097695

**Published:** 2014-05-19

**Authors:** Nadine Eckert, Florian Wrensch, Sabine Gärtner, Navaneethan Palanisamy, Ulrike Goedecke, Nils Jäger, Stefan Pöhlmann, Michael Winkler

**Affiliations:** Infection Biology Unit, German Primate Center, Göttingen, Germany; Mount Sinai School of Medicine, United States of America

## Abstract

Reporter genes inserted into viral genomes enable the easy and rapid quantification of virus replication, which is instrumental to efficient in vitro screening of antiviral compounds or in vivo analysis of viral spread and pathogenesis. Based on a published design, we have generated several replication competent influenza A viruses carrying either fluorescent proteins or Gaussia luciferase. Reporter activity could be readily quantified in infected cultures, but the virus encoding Gaussia luciferase was more stable than viruses bearing fluorescent proteins and was therefore analyzed in detail. Quantification of Gaussia luciferase activity in the supernatants of infected culture allowed the convenient and highly sensitive detection of viral spread, and enzymatic activity correlated with the number of infectious particles released from infected cells. Furthermore, the Gaussia luciferase encoding virus allowed the sensitive quantification of the antiviral activity of the neuraminidase inhibitor (NAI) zanamivir and the host cell interferon-inducible transmembrane (IFITM) proteins 1–3, which are known to inhibit influenza virus entry. Finally, the virus was used to demonstrate that influenza A virus infection is sensitive to a modulator of endosomal cholesterol, in keeping with the concept that IFITMs inhibit viral entry by altering cholesterol levels in the endosomal membrane. In sum, we report the characterization of a novel influenza A reporter virus, which allows fast and sensitive detection of viral spread and its inhibition, and we show that influenza A virus entry is sensitive to alterations of endosomal cholesterol levels.

## Introduction

Influenza A viruses are major human pathogens causing annual epidemics and intermittent pandemics with potentially devastating consequences [1]. Vaccination can protect against epidemic influenza, but needs to be repeated every year due to the constantly changing viral surface proteins [2], [3]. Therapy is available and constitutes the only defense against pandemic influenza, but its efficacy is compromised by resistance development [4], [5]. In addition, major aspects of influenza pathogenesis, such as the target cell type, are incompletely understood.

One constraint to influenza virus research is the need to quantify viral titers via time and labor intensive techniques [6]. Viruses encoding reporter genes may constitute an attractive solution to this problem. However, the insertion of reporter genes into the viral genome is a complicated task, considering that the integrity of most coding sequences is required for efficient viral spread in cell culture. In addition, coding and non-coding sequences at the ends of the genomic segments overlap with essential packaging signals and cannot be readily manipulated without interfering with the viral replicative capacity [7]. Within previous strategies, coding sequences were replaced by reporter genes. The resulting viruses were suitable for experimental analysis and screening endeavors but required the propagation in cell lines engineered to complement the missing gene [8]–[10]. Ideally, the addition of reporter genes to the genome should generate a viable and stable virus able to replicate in the same spectrum of target cells as the corresponding wild type (wt) virus. Several such replication-competent influenza A viruses carrying reporter genes have been described to date [11]–[13]. An elegant approach to the design of replication competent reporter viruses was used by Manicassamy and colleagues [14] who inserted the green fluorescent protein (GFP) reporter into segment 8 flanked by complete NS1 and NEP/NS2 genes. The genes were arranged as a single open reading frame encoding the NS1-GFP fusion protein connected to NEP/NS2 via a 2A StopGo-sequence, which terminates translation followed by re-initiation [15]. This strategy circumvented the duplication of packaging signals, but required mutation of the splice acceptor signal to prevent splicing of this segment. While this virus was used for in vivo detection of influenza A virus replication [14], GFP is not ideally suited due to green autofluorescence of tissues. Also, for quantitation in cell-based systems, enzymatic reporter assays are more sensitive.

One attractive application for influenza A reporter viruses is the analysis of viral entry into target cells, which depends on the viral hemagglutinin (HA)-driven fusion of the viral envelope with the endosomal membrane [16]. Recently, it has become clear that influenza A virus entry is a target for innate, host cell encoded antiviral defenses. Thus, the IFITM proteins 1, 2 and 3 block influenza A virus infection at the stage of viral entry and polymorphisms in the IFITM3 coding region modulate the susceptibility to disease [17], [18]. The mechanism underlying the antiviral activity of IFITM proteins is incompletely understood. A recent study proposed that IFITM1–3 increase cholesterol levels in endosomal compartments and thereby inhibit HA-driven membrane fusion [19]. Alternatively, IFITMs may impose a structural constraint on endosomal vesicles to make fusion energetically unfavorable [20].

Here, we characterize an influenza A reporter virus encoding secreted Gaussia luciferase, which enables quantification of virus infection and spread by analysis of enzymatic activity in cell culture supernatant. This virus could be used to determine EC_50_ values for the antiviral substance zanamivir and to demonstrate inhibition of influenza A virus infection by IFITM2 and 3 proteins and cholesterol modulating agents.

## Results

### Construction of a Bidirectional Vector for Golden Gate Cloning of Viral Genomic Segments

As initial step for cloning of viral genomic segments, we prepared a modified version of the reverse genetics vector pHW2000 [21], which allows bi-directional expression of genes and vRNA from cloned influenza virus segments. We made two modifications to this vector. First, we chose to use AarI restriction sites for cloning instead of the BsmBI sites, since an analysis of several laboratory and seasonal strains of influenza A virus had shown that AarI but not BsmBI rarely cuts within segments. Second, we inserted a cassette with the chloramphenicol resistance gene and the negative selection marker *ccdB* between the AarI sites. The *ccdB* gene acts as a toxin that kills *E. coli* by interfering with DNA gyrase activity [22], [23]. This setup allows cloning of inserts in one-pot restriction-ligation reactions, called Golden Gate cloning [24]. The vector pHW2000-GGAarI was constructed by restriction free cloning [25] and propagated in *E. coli* DB3.1 bacteria, which are resistant to *ccdB* due to a mutation in the DNA gyrase gene.

### Construction and Reconstitution of Reporter Viruses

**Figure 1:**
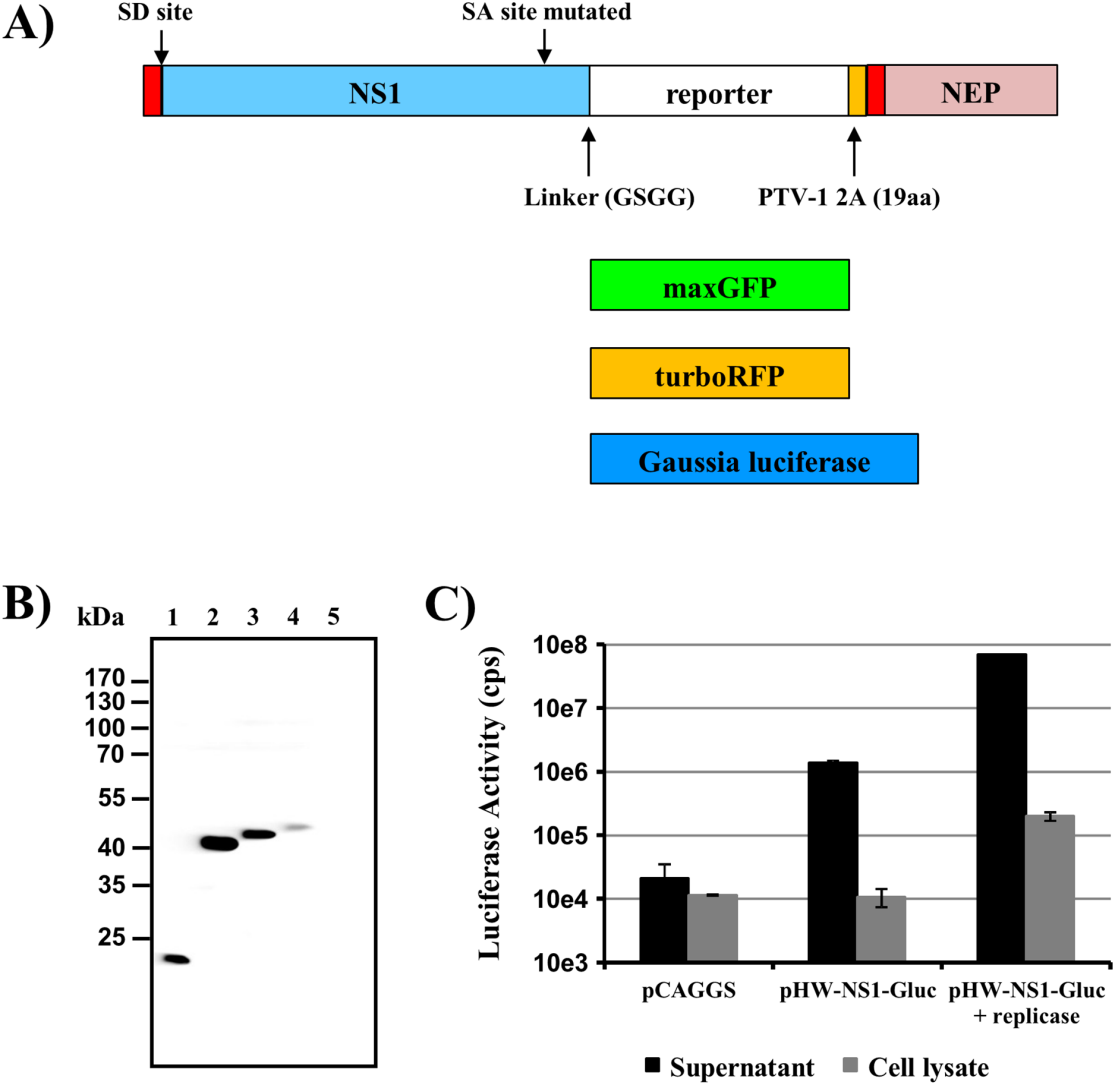
Construction of reporter viruses. (A) Overview of the design of genomic segments containing reporter genes. We used a previously reported design [14], where complete NS1 and NEP/NS2 genes flank a reporter gene. Additional features such as a Gly-Ser-Gly-Gly linker between NS1 and reporter gene and the porcine teschovirus 1 2A “StopGo” sequence are indicated, as are the splice donor and the mutated splice acceptor site. The reporter genes used are indicated below. (B) 293T cells were transfected with pHW2000-based plasmids carrying either wild type segment 8 (lane 1) or segments modified with GFP (lane 2), turbo RFP (lane 3) or Gaussia luciferase (lane 4). Cells transfected with empty vector served as control (lane 5). NS1 fusion proteins were detected by immunoblot with an antibody directed against NS1. (C) 293T cells were transfected with control plasmid (pCAGGS), pHW-NS1-Gluc or pHW-NS1-Gluc and plasmids encoding replicase proteins. At 24 h post transfection, supernatants and cell lysates were analyzed for Gaussia luciferase activity. The results of a representative experiment performed with triplicate samples are shown; error bars indicate standard deviation (SD). Similar results were obtained in a separate experiment. cps, counts per second.

We decided to generate reporter viruses based on a published design [14], where the reporter gene had been fused to the NS1 gene in segment 8. The whole construct was assembled by PCR as described in the initial report and cloned into pHW2000. As additional reporter genes, we chose turboRFP and Gaussia luciferase, which were inserted instead of GFP by restriction free cloning (Fig. 1A).

To assess expression of the NS1-fusion proteins, we transfected all newly constructed plasmids and a plasmid containing wild type segment 8 of A/PR/8/34 into 293T cells and analyzed the expressed proteins by immunoblot. All proteins were expressed and showed signals of the expected size (Fig. 1B). The absence of a band corresponding to a NS1-Gaussia luciferase-NS2/NEP fusion protein demonstrated that the 2A “StopGo” signal was effective. The amount of the NS1-Gaussia luciferase fusion protein in the cell lysates was low compared to wild type NS1 protein and the other fusion proteins. Since Gaussia luciferase is secreted from cells, we assessed whether the same holds true for the NS1-Gaussia fusion protein. For this, we analyzed luciferase activities from supernatants and cell lysates of 293T cells transfected with control plasmid, the Gaussia reporter construct or the Gaussia reporter construct along with plasmids encoding influenza A replicase proteins. As shown in Fig. 1C, high amounts of Gaussia luciferase activity were detected only in culture supernatants, with the highest activity measured in supernatants of cells expressing segment 8 jointly with the viral replicase proteins, demonstrating that virtually all Gaussia luciferase is secreted [26].

**Figure 2:**
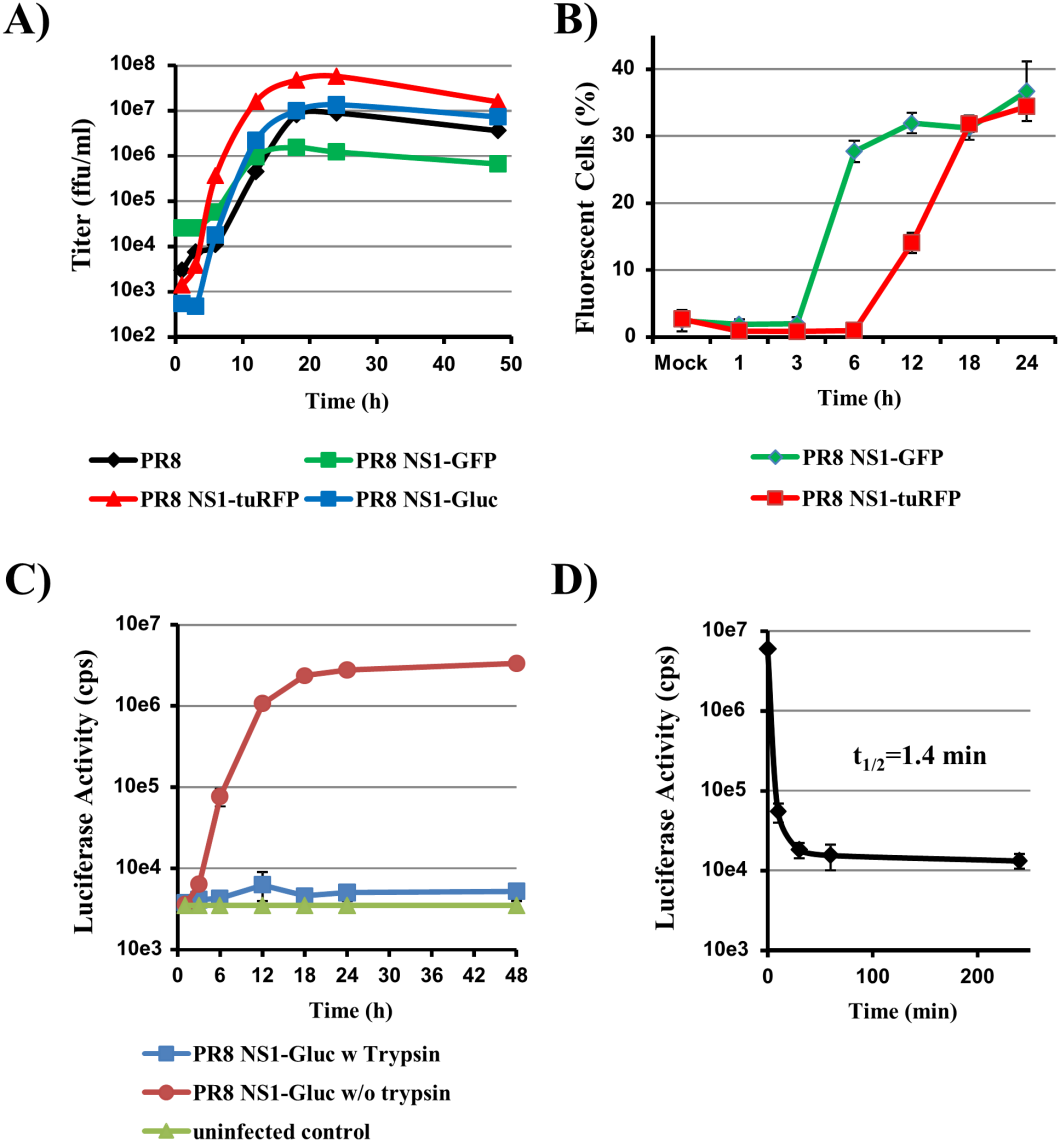
Growth and expression characteristics of reconstituted reporter viruses. (A) Single step growth curves were determined on MDCK cells infected at an MOI of 1 with parental PR8 virus or reporter viruses PR8-NS1-GFP, PR8-NS1-tuRFP and PR8-NS1-Gluc. (B) Kinetics of fluorescent reporter gene expression. MDCK cells were infected with PR8-NS1-GFP and PR8-NS1-tuRFP at MOI 1. Cells were harvested at the indicated time points after infection and analyzed by flow cytometry. (C) Kinetics of Gaussia activity in the supernatants of infected and uninfected cells. MDCK cells were infected with PR8-NS1-Gluc at MOI 1 and cultivated in the presence and absence of trypsin. Supernatants were collected, frozen and measured together. (D) Supernatant containing high Gaussia luciferase activity was incubated with trypsin (1 µg/ml). Luciferase activity was measured at the indicated intervals. The calculated half-life of luciferase activity is indicated. All panels show the results of a representative experiment performed with triplicate samples; error bars indicate SD. Similar results were obtained in a separate experiment. cps, counts per second.

All three segments encoding the different reporter genes (GFP, turboRFP and Gaussia luciferase) allowed reconstitution of infectious virus in the context of A/PR/8/34 (PR8). Growth of the three viruses PR8 NS1-GFP, PR8 NS1-tuRFP and PR8 NS1-Gluc was analyzed in single step growth curves on MDCK cells infected at a multiplicity of infection (MOI) of 1. After a lag phase of 3 hours, virus titers in the supernatant rapidly increased for all viruses and reached a plateau at 18 hours post infection (hpi) (Fig. 2A). When compared with wild type PR8, the differences in the titers of all viruses were within 1 log, demonstrating that insertion of the reporter genes was compatible with rapid virus growth.

Next, we analyzed the kinetics of reporter gene expression. MDCK cells were infected with reporter viruses carrying fluorescent reporter genes with MOI 1, harvested at fixed time points and analyzed by flow cytometry (Fig. 2B). Green fluorescence emitted by PR8 NS1-GFP was detected as early as 6 hpi, whereas red fluorescence of PR8 NS1-tuRFP could be detected at 12 hpi reaching maximum levels at 18 hpi. For both viruses a maximum of 30–35% of cells were infected. To analyze kinetics of Gaussia luciferase expression, we infected MDCK cells with PR8 NS1-Gluc and incubated the cells in the presence and absence of trypsin, which is required for HA activation and thus multi-cycle replication of influenza A viruses [27]. Significant amounts of luciferase activity were detected as early as 6 hpi and reached a maximum at 18 hpi (Fig. 2C). Luciferase activity was only detected in supernatants from cells cultivated in the absence of trypsin, suggesting that Gaussia luciferase might be inactivated by trypsin. Indeed, addition of trypsin completely abrogated Gaussia luciferase activity in culture supernatants (Fig. 2D), in agreement with observations on other luciferases [28]. Thus, single cycle infection of cells with PR8 NS1-Gluc can be easily detected by measuring Gaussia activity in culture supernatants, while experiments assessing viral spread must be conducted in the presence of an HA-activating protease which does not impact Gaussia activity. The membrane-associated HA activating protease TMPRSS2 [29], [29,30] might be one suitable option.

### Genetic Stability of Reporter Viruses

**Figure 3:**
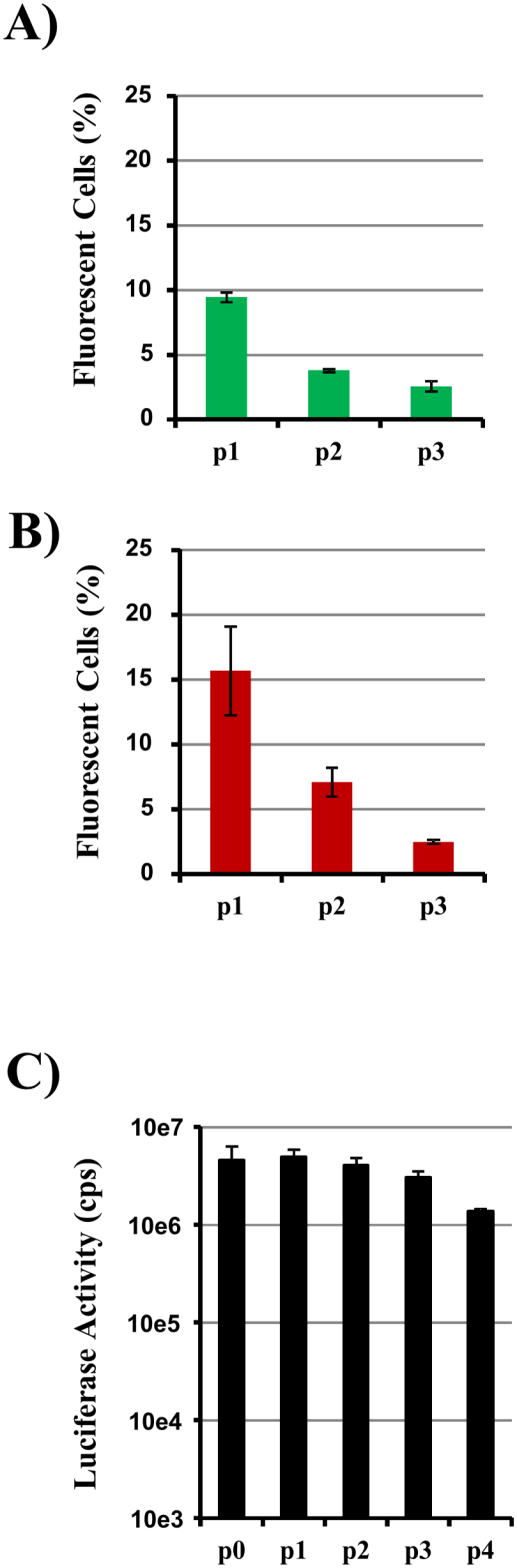
Stability of reporter viruses. Viruses PR8-NS1-GFP (A), PR8-NS1-tuRFP (B) and PR8-NS1-Gluc (C) were passaged in MDCK cells. Stocks generated in this way were used to infect MDCK cells at MOI 1. Reporter gene activity was determined by flow cytometric analysis of infected cells (A, B) or by measurement of luciferase activities present in culture supernatants at 24 h post infection (C). All panels show the results of a representative experiment performed with triplicate samples; error bars indicate SD. Similar results were obtained in a separate experiment. cps, counts per second.

We next investigated the stability of PR8-NS1-GFP, PR8-NS1-tuRFP and PR8-NS1-Gluc by comparing reporter gene activity when cells were infected with viruses from different passages at the same MOI. Both fluorescent viruses, PR8-NS1-GFP (Fig. 3A) and PR8-NS1-tuRFP (Fig. 3B), showed a decrease in the percentage of fluorescent cells down to a few percent in passage 3. In contrast, the virus carrying Gaussia luciferase was relatively stable and loss in activity could only be observed after passage 4. However, at this point the Gaussia luciferase activity in infected cultures was still 2–3 log above background. Due to this high sensitivity and the higher stability of PR8-NS1-Gluc relative to PR8-NS1-GFP and PR8-NS1-tuRFP, we used the reporter virus encoding Gaussia luciferase for all of the further experiments.

### The Gaussia Reporter Virus is Suitable for Antiviral Drug Testing

**Figure 4:**
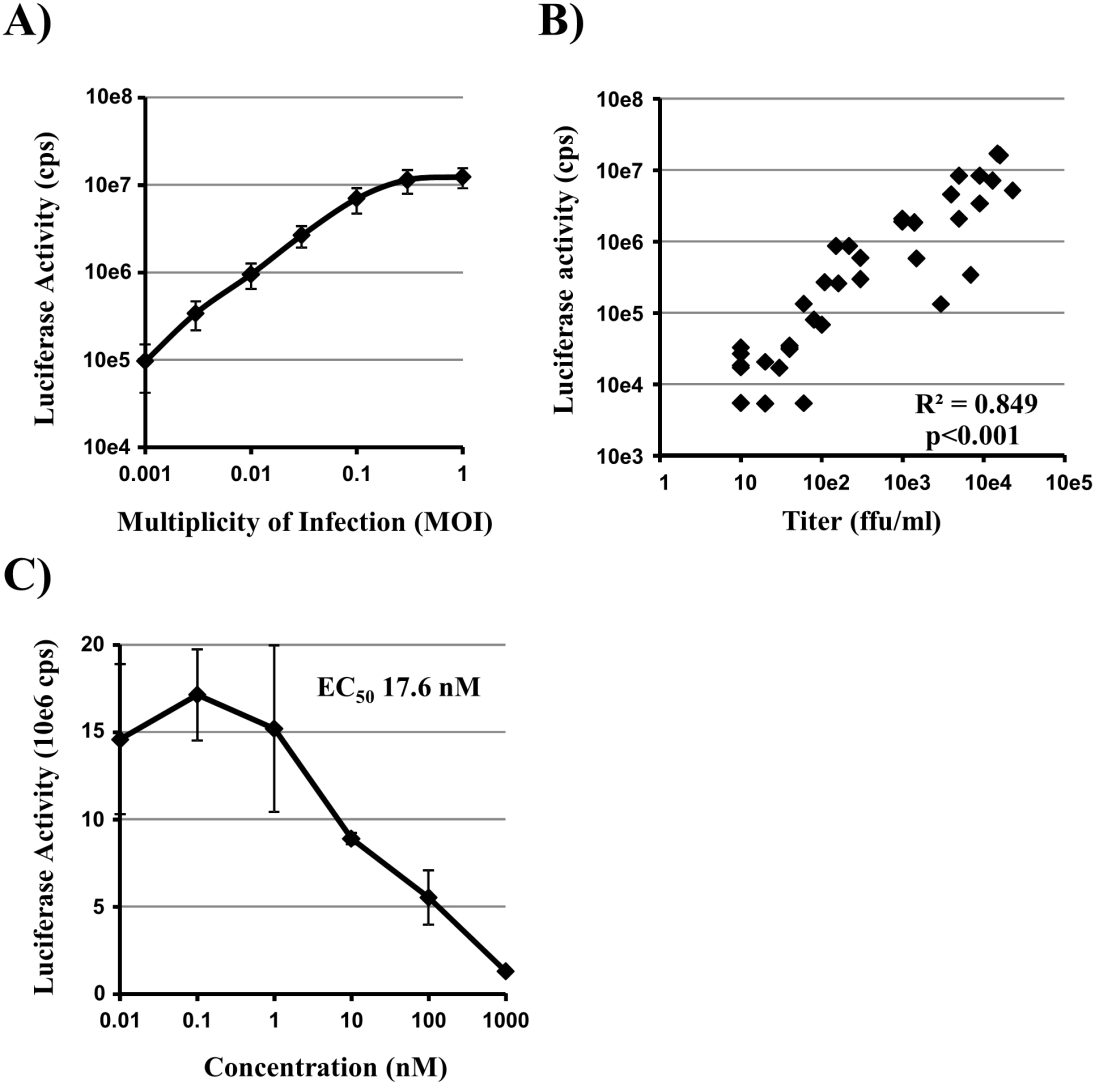
Gaussia luciferase reporter virus is suitable to measure the antiviral activity of chemical compounds. (A) MDCK cells were infected with PR8-NS1-Gluc at the indicated MOIs. Luciferase activity was determined in supernatants harvested at 24 h post infection. Error bars indicate SD of triplicate samples. Similar results were obtained in a separate experiment. (B) Caco-2 cells were infected in 6-well plates with 100 ffu. Supernatants were collected over the course of three days and luciferase activity and virus titer was determined for each sample. Results of three independent experiments performed in triplicates are shown. The R^2^ and p values of the linear regression analysis are shown in the graph. (C) Caco-2 cells were infected in 6-well plates with MOI 0.004. After infection, cells were incubated with the indicated concentrations of zanamivir. Gaussia luciferase activity was determined at 52 h post infection. Error bars indicate SD of triplicate samples. Calculated effective concentration EC_50_ is indicated. Similar results were obtained in three separate experiments. cps, counts per second.

The high sensitivity and the simplicity of measuring Gaussia luciferase activity prompted us to ask whether this virus could be used for inhibitor testing and screening purposes. We first tested the relation of Gaussia luciferase activity and the MOI of input virus. As demonstrated in Fig. 4A, we observed a log-linear correlation between MOI and Gaussia luciferase activity over more than 2 log of infectious dose, which leveled off roughly at an MOI of 0.3. In addition, we analyzed, if we can observe a similar correlation between luciferase activity and output virus titers. For this, we chose an experimental setup measuring multi-cycle replication. Since we could not ensure activation of influenza HA by addition of trypsin to cultures, we opted to infect CaCo-2 cells, which allows for trypsin-independent influenza virus spread [30], [31] due to endogenous expression of TMPRSS2 [30]. After infection of cells with a low dose of virus (100 ffu, MOI 0.0004) supernatants were collected at several time points up to three days post infection. For each sample luciferase activity and virus titer were determined. As shown in Fig. 4B we observe a good correlation between Gaussia luciferase activity and virus titer over more than 2 log. Thus, we conclude from both experiments, that this virus is suitable for analyses over a wide range of infectious doses.

Accordingly, we analyzed the applicability of this virus for the screening of antiviral substances. Since drugs currently in clinical use target neuraminidase (NA) and act on virus release, we again chose a multi-cycle setup. Caco2 cells were infected with a low dose of virus (1000 ffu, MOI 0.004), treated with varying concentrations of NA inhibitor zanamivir and 3 days post infection Gaussia luciferase activity was determined in the supernatants. Dose response curves showed clear inhibition by zanamivir (Fig. 4C). The EC_50_ value was calculated to 17 nM, which is in the range known from other studies [32]–[34]. Therefore, this virus is suitable for in vitro testing of antiviral activity of zanamivir and most likely other antiviral compounds.

### The Antiviral Activity of IFITM Proteins can be Determined with the Gaussia Reporter Virus

**Figure 5:**
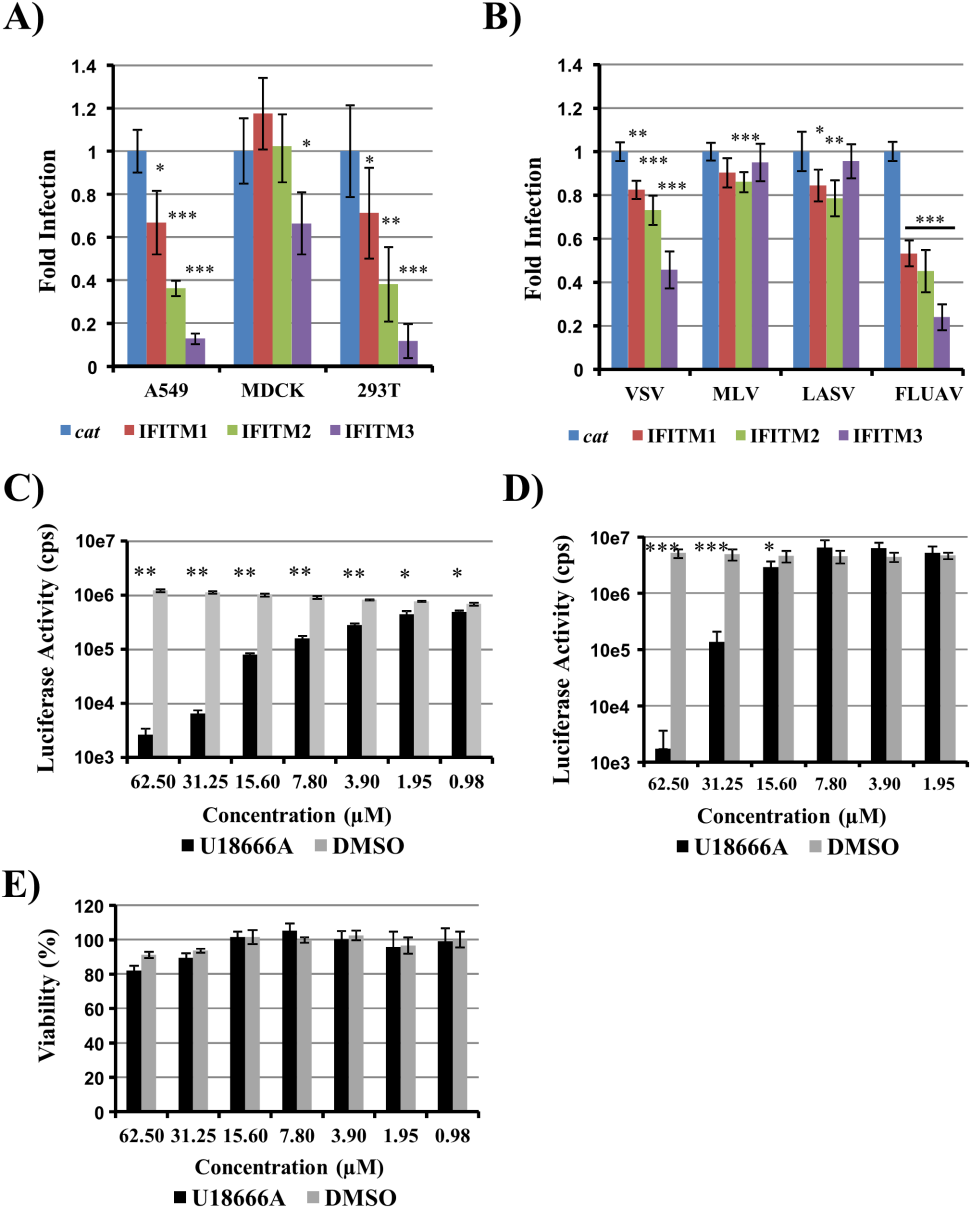
Gaussia luciferase reporter virus is suitable to measure inhibitory effects by IFITM proteins. (A) A549, MDCK and 293T cells were transduced with vectors encoding IFITM1, 2 or 3 or *cat* as control gene and subsequently infected with PR8-NS1-Gluc at MOI 0.1. Gaussia luciferase activity was determined from supernatants at 24 h post infection. The average of two (MDCK, 293T) or three (A549) separate experiments performed with triplicate samples is shown. Infection of cat expressing control cells was set as 100%. Error bars indicate SEM. (B) 293T cells transduced to express IFITM1, 2 or 3 or *cat* were transduced with reporter pseudoparticles bearing the surface proteins of VSV, MLV, LASV or influenza A virus (FLUAV). Firefly luciferase activity encoded by pseudovirus genomes was determined in cell extracts harvested at 72 hours after transduction. The average of three separate experiments performed with triplicate samples is shown. Infection of *cat* expressing control cells was set as 100%. Error bars indicate SEM. (C) A549 cells were infected with PR8-NS1-Gluc at MOI 0.1 in the presence of the indicated concentrations of cholesterol modulating agent U18666A. Gaussia luciferase activity in culture supernatants was determined at 24 hours post infection. (D) A549 cells were incubated with the indicated concentrations of U18666A and then transduced with pseudoparticles bearing influenza A virus HA and NA proteins on the surface. Firefly luciferase activity encoded by pseudovirus genomes was determined in cell extracts harvested at 72 h post transduction. (E) Viability of A549 cells treated with U18666A at the indicated concentrations or diluent DMSO was measured using MTT test. Panels C-E show the results of a representative experiment performed with triplicate samples; error bars indicate SD. Similar results were obtained in at least two separate experiments. All results were assessed for statistically significant differences using a two-tailed t test: *, P≤0.05; **, P≤0.01; ***, P≤0.001. cps, counts per second; VSV, vesicular stomatitis virus; MLV, murine leukemia virus; LASV, Lassa virus; FLUAV, influenza A virus.

Numerous natural antiviral factors are expressed upon interferon induction. Members of the IFITM protein family, specifically IFITM1, 2 and 3, have been identified as inhibitors of influenza A virus infection [17]. We used these proteins to determine whether the PR8-NS1-Gluc virus is suitable to quantify influenza A virus inhibition by cellular proteins. For this, we transduced A549, MDCK and 293T cells with vectors encoding IFITM 1, 2 or 3 or chloramphenicol acetyltransferase (*cat*) as a control gene. Subsequently, we infected the transduced cells with PR8-NS1-Gluc at an MOI of 0.1 in the absence of trypsin and determined Gaussia luciferase activity in culture supernatants. Expression of IFITM3 resulted in marked inhibition of PR8-NS1-Gluc infection in all cellular systems tested (Fig. 5A), in keeping with published results [17], [35]. Robust inhibition of infection was also observed upon IFITM2 expression in A549 and 293T cells but not in MDCK (Fig. 5A), potentially due to differences in expression efficiency. In contrast, the inhibitory effects observed upon expression of IFITM1 were minor (A549 and 293T cells) or absent (MDCK cells), again in agreement with published data [17], [35]. Similar effects were measured upon transduction of IFITM protein expressing 293T cells with a retroviral vector bearing the HA and NA proteins of A/WSN/33 (Fig. 5B). Thus, strongest inhibition was detected upon directed expression of IFITM3 while the antiviral activity of IFITM2 and particularly IFITM1 was less pronounced. A similar but less pronounced inhibition was observed for pseudoparticles bearing the vesicular stomatitis virus (VSV) glycoprotein on their surface, while no inhibition was measured for pseudoparticles bearing murine leukemia virus envelope protein (MLV) or the Lassa virus (LASV) glycoprotein, in keeping with published data [17]. In sum, these results indicate that PR8-NS1-Gluc can be used to determine the antiviral activity of host cell proteins.

### Alteration of Endosomal Cholesterol Levels Inhibits Influenza a Virus Entry

Although the antiviral activity of IFITM proteins has been clearly demonstrated in cell culture [17], [35] and in the infected host [18], [36], the mechanisms underlying the antiviral action is less clear. Recently, it was proposed that IFITM proteins might impede viral entry by increasing the cholesterol levels in the endosomal compartment [19]. To investigate this possibility, we tested whether U18666A, a compound which increases endosomal cholesterol [37], inhibits influenza A virus entry. Inhibition of PR8-NS1-Gluc infection by U18666A was observed at all concentrations tested. The inhibitory effects were dose dependent and particularly pronounced at U18666A concentrations above 7.8 µM (Fig. 5C). These results were reproduced with pseudoparticles bearing influenza HA and NA (Fig. 5D), confirming that viral entry was inhibited. Finally, inhibitory concentrations up to 62.5 µM were nontoxic to A549 cells (Fig. 5E), indicating that the antiviral effects observed were specific. Thus, modulation of endosomal cholesterol levels can impede influenza A virus infection.

## Discussion

We generated Influenza A viruses carrying three different reporter genes, based on a design published by Manicassamy and colleagues [14]. Two recombinant viruses encoded the fluorescent proteins GFP and turbo RFP, while a third virus encoded Gaussia luciferase. Red fluorescent proteins should be advantageous for localization studies in tissues or organ cultures, where green auto-fluorescence is often a problem. The virus carrying the secreted Gaussia luciferase should allow sensitive detection of infection without the requirement to lyse infected cultures. For initial cloning of the constructs we generated a modified version of pHW2000, which is suitable for cloning in one-pot restriction-ligation reactions, called Golden Gate cloning [24]. In our hands the cloning efficiency of pHW2000-GGAarI and pHW2000 was comparable, but pHW2000-GGAarI was easier and faster to use, since no additional steps for vector preparation were needed. Recently, a similar vector was published, which allowed efficient cloning of influenza A virus genomes [38]. The NS1-reporter fusion proteins expressed from the modified genomic segments were of the expected size and Gaussia luciferase activity was readily detectable in the supernatant of transfected and infected cells.

All reporter viruses could be reconstituted and grew like wild type virus in single step growth curves. Maximum levels of reporter protein activity were measured at 18 hpi for all reporter viruses. Fluorescence was detected significantly earlier in cultures infected with PR8-NS1-GFP relative to PR8-NS1-tuRFP. This might be explained with the faster maturation rate of GFP compared to turbo RFP [39]. Gaussia luciferase activity was only detected when no trypsin was present in the medium; in the presence of trypsin the half-life of Gaussia luciferase was very short. Therefore, experimental conditions which do not require addition of trypsin to the medium for influenza A virus activation need to be established. However, single cycle experiments, as carried out in the present study, can be readily conducted in the absence of trypsin. In fact, single cycle experiments carried out with PR8-NS1-Gluc virus stocks produced in the presence of trypsin benefit from the lack of Gaussia luciferase activity in the viral inoculum. When multiple rounds of virus replication need to be monitored, as in the measurement of the antiviral activity of zanamivir, it is necessary to use cell lines that express activating proteases like TMPRSS2, such as CaCo-2 [30] or MDCK-TMPRSS2 cells [29].

Upon passaging of the reconstituted viruses, we observed limited genetic stability of the viruses encoding fluorescent proteins. Here, PR8-NS1-tuRFP seemed to be slightly more stable than PR8-NS1-GFP. In contrast, PR8-NS1-Gluc was quite stable during the first four passages and only showed reduced reporter activity at later passages. Instability has also been reported for a virus encoding a NS1-GFP fusion protein and lacking part of the NS1 sequence [40]. Reasons for the genetic instability are currently not clear. There are two duplicate regions in the genome configuration used, one in exon 1 of the NS genes and one in exon 2 up to the stop codon of NS1, which might be susceptible to RNA recombination. Moreover, it is not clear why the virus encoding Gaussia luciferase seems to be more stable than the fluorescent viruses. However, upon prolonged passaging we also observed a reduction in luciferase activity for this virus.

Recently, a different influenza A virus carrying Gaussia luciferase was described and used for in vitro and in vivo characterization of broadly neutralizing antibodies [41]. In this virus, which exhibited stability comparable to PR8-NS1-Gluc, Gaussia luciferase was connected via a 2A sequence to PB2 and had a KDEL signal for retention in the endoplasmic reticulum. Because of this retention signal, the luciferase remained inside the cells and lysis of cells was required to measure Gaussia luciferase activity. This virus, as well as recently documented influenza reporter viruses [42], [43], is suitable for in vivo experiments. However, the secretion of Gaussia luciferase from cells infected with the PR8-NS1-Gluc described in the present study is of significant advantage in medium to high throughput in vitro assays, which are a prerequisite to efficient screening of compound libraries for novel antivirals.

For use in the quantification of viral infectivity and spread, a good correlation between infectious dose and measured values is necessary. We observed a good correlation of Gaussia luciferase activity and both input virus (MOI) or output virus over more than two orders of magnitude of infectious dose, making this system ideal for quantitative measurements of virus infection and its inhibition by antiviral compounds. This was demonstrated by measuring the effectiveness of zanamivir, a drug currently in clinical use which targets the viral NA. Employing CaCo-2 cells, we measured EC_50_ values between 10–20 nM. These values are close to those previously reported for other influenza A virus strains tested in different cellular systems [32]–[34]. We note as a caveat that enzymatic NA assays are better suited than virus growth assays to predict antiviral activity of NAI in the infected host [44], [45]. This might be a consequence of the extracellular mode of action of NA. However, our results demonstrate that the virus expressing Gaussia luciferase is able to faithfully replicate observations made in other systems with authentic influenza A virus and will be a valuable tool for future identification and characterization of antiviral compounds.

Cells encode several antiviral factors whose expression can be induced by interferons [46]. Recently, the proteins IFITM1, 2 and 3, the expression of which can be induced by type I interferons [47], [48], have been described to possess potent antiviral activity in vitro and in vivo [17], [20]. So far most of the studies analyzing the antiviral action of IFITM proteins in cell culture have relied on the use of pseudoparticles with important findings being reproduced with wild type influenza A virus. We confirmed these results with the Gaussia luciferase based influenza virus system by demonstrating that IFITM2 and 3 block influenza A virus infection.

Recently, it has been suggested that IFITM proteins might exert antiviral activity by increasing cholesterol levels in endosomal compartments [19]. While U18666A can increase endosomal cholesterol levels (2–3-fold increased in size of compartments) [19], the potential of U18666A to inhibit influenza A virus infection is currently unknown. We found that U18666A inhibits influenza A virus infection at the stage of viral entry. The U18666A effect on cholesterol is similar to mutation of the Niemann-Pick C1 (NPC1) cholesterol transporter [37]. Ebola virus entry into host cells depends on binding of the proteolytically processed viral glycoprotein to NPC1 [49], [50] and entry can be inhibited by U18666A [49], [51]. In contrast, influenza A virus (PR8) can infect haploid human cell lines deficient in NPC1 [49]. These observations suggest that Ebola but not influenza virus entry requires NPC1 expression while entry of both viruses might depend on normal endosomal cholesterol levels.

In summary, we constructed a recombinant influenza A virus encoding Gaussia luciferase, which allows sensitive quantification of infection with minimal manipulation. This virus is genetically stable over several passages and suitable for analysis of the antiviral action of cellular factors or chemical compounds and will be very helpful in the screening and evaluation of novel antiviral compounds.

## Materials and Methods

### Plasmids

The eight plasmid system for low and high virulent strains A/lvPR/8/34 (H1N1) or A/hvPR/8/34 (H1N1) and expression plasmids for influenza A/Thai/1(Kan-1)/04 replicase (PB1, PB2, PA and NP in pCAGGS) were described previously [52], [53].

An improved version of pHW2000 [21], [54] was generated by restriction free cloning [25]. We made two modifications to the vector. First, we chose to use AarI for cloning instead of BsmBI in the original vector, since an analysis of several laboratory and seasonal strains of influenza A virus had shown that AarI rarely cuts within segments. Second, we inserted a cassette with the chloramphenicol resistance gene and the negative selection marker *ccdB* between the AarI sites. The *ccdB* gene acts as a toxin that kills *E. coli* by interfering with DNA gyrase. This setup allows cloning of inserts in one pot restriction-ligation reactions, called Golden Gate cloning [24]. In this reaction, the desired cloning product lacks the recognition sites for the restriction enzyme. Religation of the *cat*-*ccdB* cassette can occur, but will be cleaved again or negatively selected after transformation in conventional *E. coli* strains. For construction of pHW2000-GGAarI, we first amplified a fragment containing chloramphenicol resistance and *ccdB* from pENTR-2B-DUAL (Invitrogen, Germany) using primers HM2000GGAar-RF3 CGAGTCGGCATTTTGGGCCGCCGGGTTATTGATCGCAGGTGCTGGCTGTGTATAAGGGAGCC and HM2000GGAar-RF5 GTACTGGTCGACCTCCGAAGTTGGGGGGGAGATCGCAGGTGTTAGGCACCCCAGGCTTTACAC. The purified fragment was used in a second PCR reaction using pHW188-NS as template to add the pHW2000 vector backbone. After DpnI digestion the DNA was transformed into *E. coli* DB3.1 and selected on ampicillin and chloramphenicol. The DB3.1 strain is resistant to *ccdB* due to a mutation in the DNA gyrase gene. The resulting plasmid was confirmed by restriction digest and sequencing.

A plasmid encoding segment 8 from strain A/PR/8/34 New York (kindly provided by Stephan Ludwig) was generated after RT-PCR amplification from virion RNA using primers fluA AarI-NS-1 5- CGATCACCTGCTCGAGGGAGCAAAAGCAGGGTG-3 and fluA AarI-NS-890R 5- CGATCACCTGCTCTCTATTAGTAGAAACAAGGGTGTTTT-3, and subsequent Golden Gate cloning [24] into pHW2000-GGAarI.

The plasmid containing the NS1-GFP-2A-NEP fusion construct described by [14] was generated after RT-PCR amplification from virion RNA using primers fluA AarI-NS-1 5- CGATCACCTGCTCGAGGGAGCAAAAGCAGGGTG-3 and fluA AarI-NS-890R 5- CGATCACCTGCTCTCTATTAGTAGAAACAAGGGTGTTTT-3, and cloning into pHW2000-GGAarI. From this starting construct, we generated plasmids encoding turboRFP or Gaussia luciferase in place of GFP by restriction free cloning [25] using primers rfc-NS1g-tuRFP5pr 5- GAAATGGCGGGAACAATTAGAAGCGAAGTTGGGTCCGGCATGAGCGAGCTGATCAAGGAG-3, rfc-NS1g-tuRFP3 5- CGCCTGTTTCAGCAGGCTAAAGTTGGTCGCGCCGCTGCCTCTGTGCCCCAGTTTGCTAGG-3, rfc-NS1g-Gluc5pr 5- GAAATGGCGGGAACAATTAGAAGCGAAGTTGGGTCCGGCATGGGAGTCAAAGTTCTGTTTG-3 and rfc-NS1g-Gluc3 5- CCGCCTGTTTCAGCAGGCTAAAGTTGGTCGCGCCGCTGCCGTCACCACCGGCCCCCTTGATC-3. As templates, we used the plasmids pLemiR (Open Biosystems) and pCMV-GLUC-1 (PJK, Germany).

Retroviral vectors for expression of IFITM1, 2 and 3 have been described previously [17], [55]. A version of pQCXIP-IFITM2 with correct stop codon was amplified with IFITM2-5NA 5-CCCGCGGCCGCACCGGTACCATGAACCACATTG-3 and IFITM2-3BE 5-CGAATTCCGGATCCCTATCGCTGGGCCTGGAC-3 and cloned in pQCXIP using NotI and EcoRI. For construction of the control vector pQCXIP-Cat, pENTR-2B-Cat was digested with NotI and BglII and ligated to pQCXIP digested with NotI and BamHI. The *cat* gene had been amplified with primers ENTRcat3Xho and ENTRcat5Acc and cloned into pENTR-2B-Dual (Invitrogen, Germany) via Acc65I and XhoI. Plasmids for viral envelope proteins from Vesicular stomatitis virus (VSV), Mouse leukemia virus (MLV) and Lassa virus (LASV) have been described previously [56].

### Cell Culture

We used minimum essential medium with Earle’s salts (MEM, PAA) for cultivation of MDCK II and CaCo-2 cells, and Dulbecco's modified Eagle’s medium (DMEM; Invitrogen) for culture of 293T and A549 cells. All media were supplemented with 10% fetal calf serum (FCS), glutamine, penicillin and streptomycin, and all cell cultures were maintained at 37°C under a 5% CO_2_ atmosphere.

### Luciferase Assay

We used D-PBS (with Ca and Mg) as assay buffer to measure Gaussia luciferase activity in the supernatant of infected cells [26]. Coelenterazine was added from a stock solution (in acidified ethanol) to a final concentration of 1.5 µM. To measure Firefly luciferase activity, cells were harvested in Promega Lysis Reagent (25 mM Tris-phosphate pH 7.8, 2 mM DTT, 2 mM 1,2-diaminocyclohexane-N,N,N′,N′-tetraacetic acid, 10% glycerol, 1% Triton X-100). Measurements were taken in a Hidex Plate Chameleon V instrument with 100 ms delay and 2 s counting time.

### Immunoblot

For immunoblotting, cell lysates were separated by SDS-PAGE and blotted onto nitrocellulose membranes. Membranes were blocked with 5% milk powder in PBS/0.1% Tween. NS1 proteins were detected using an antibody against NS1 protein from A/California/06/2009 (H1N1) [57]. Bound antibodies were detected with horseradish peroxidase-(HRP-)-conjugated secondary antibody (Dianova) at a dilution of 1∶5000 and reacted with a luminescent substrate (Amersham ECL reagent, GE Healthcare, München).

### Production of Influenza Virus

A 1∶1 mixture of 293T and MDCK II cells was seeded at a density of 2.5×10^5^ cells/well in 6-well plates the day before transfection. At the following day, cells were transfected by calcium phosphate co-precipitation with 1 µg of each plasmid from the eight plasmid system. Cells were incubated in DMEM/10% FCS for two days and then placed in infection medium, MEM containing 0.1% bovine serum albumin (BSA) and 5 µg/µl TPCK-trypsin. Supernatant was harvested from day 4 to 10 after transfection and virus titer determined by focus formation assay [6], [30].

Virus stocks (passage 1 and higher) were generated by inoculation of 3 million MDCK II cells in T75 flasks with 10 µl virus supernatant in 2 mL infection medium supplemented with 5 µg/mL TPCK-trypsin. After infection, cells were incubated in a total volume of 10 mL infection medium with trypsin. Supernatant was harvested after about 24 h, when most of the cells were detached.

For single step growth kinetics, cells were seeded in 48-well plates (10^5^ cells/well). On the next day, cells were infected with MOI 1 for 1 h at 37°C. After washing with PBS, cells were incubated in infection medium with or without TPCK-trypsin (1 µg/mL). At certain time points supernatant was harvested and frozen at −80°C. After completion, virus titers were determined for all samples by focus formation assay.

### Virus Titration by Focus Formation Assay

Virus titers were determined by focus formation assay [6], [30]. Briefly, MDCK cells were seeded in 96-well plates at 3×10^4^ cells/well. On the next day, cells were washed once in infection medium (MEM, 0.1% BSA) without trypsin and once with PBS before 50 µl of tenfold dilutions of virus supernatant was added. After incubation for 1 h at 37°C and removal of the inoculum, 100 µl DMEM containing 1% Avicel (FMC BioPolymer) and 2 µg/mL TPCK-Trypsin (Sigma) were added and the plate incubated overnight. For detection of virus infected cells, first the Avicel containing medium was removed, then cells were washed 3–4 times with PBS and fixated with 4% paraformaldehyde. After washing, quenching (PBS, 0.5% Triton, 20 mM Glycin) and blocking (PBS, 0.5% Triton, 1% BSA), cells were incubated with primary polyclonal goat antibody raised against influenza A virus (1∶1000, Millipore) and secondary anti-goat HRP-conjugated antibody (1∶1000, KPL). All incubation steps were followed by washing steps. Finally, cells were reacted with HRP-substrate (True Blue; KPL) until blue spots became visible. Foci were counted and viral titers calculated as focus forming units (ffu).

### Infection Experiments with Influenza Viruses

For correlation of luciferase activity and output virus, and for measurement of zanamivir sensitivity, CaCo-2 cells were seeded in 6-well plates at 2.5×10^5^ cells/well. On the next day, cells were infected with 100 ffu/well for 1 h at 37°C. Cells were then incubated in medium without trypsin and supernatants were harvested and frozen at −80°C every 8–16 h for three days. From each sample luciferase activity and virus titer were determined. Linear regression analysis was performed in Microsoft Excel and R^2^ and p-values were calculated with the Excel addin Daniel’s XL Toolbox (version 6.52 http://xltoolbox.sourceforge.net/).

For analysis of zanamivir sensitivity, CaCo-2 cells were infected with 1,000 ffu/well for 1 h at 37°C. After washing with PBS, medium containing zanamivir (Sigma Aldrich) was added to the cells. Zanamivir was diluted in medium from a 10 mM stock solution to final concentrations of 1 µM, 100 nM, 10 nM, 1 nM, 0.1 nM, 0.01 nM. Supernatant was harvested after 3 days and Gaussia luciferase activity was determined. EC_50_ values were calculated using Graphpad Prism software.

The cholesterol modulating agent U18666A (Cayman Chemical Company, USA) was dissolved in DMSO. To measure inhibition of influenza A virus infection by U18666A, A549 cells were seeded in 96-well plates at 2×10^4^ cells/well. On the next day, cells were incubated for 1 h with DMSO as control or U18666A at concentrations 2-fold higher than the final concentrations obtained upon addition of virus. Then, an equal volume of medium containing virus was added. Supernatant was harvested after 24 h and Gaussia luciferase activity was measured.

Cells transduced with retroviral vectors were infected 24 h after completion of transduction with influenza PR8 NS1-Gluc at MOI 0.1 for 1 h at 37°C. Thereafter, medium was replaced by infection medium without trypsin. Supernatant was harvested after 24 h and Gaussia luciferase activity was measured.

### Determination of Cell Viability

Cell viability in the presence of U18666A or DMSO was determined using a 3-(4,5-dimethylthiazol-2-yl)-2,5-diphenyltetrazolium bromide (MTT) based cell viability assay [58]. Briefly, 2×10^4^ cells/well were pre-incubated with inhibitor at the same concentrations as used for the infection experiment. After 1 h, an equal volume of medium was added to obtain final inhibitor concentrations and the cells were incubated for 24 h. Then, medium was changed and 10 µl (1/10 vol) of MTT stock solution were added. After incubation for 4 h at 37°C in a humidified atmosphere, formazan salts were dissolved by addition of 100 µl acidified isopropanol. Absorption at 595 nm was measured in an ELISA plate reader. Absorption of untreated cells was set as 100%.

### Production of Retroviral Vectors

For production of retroviral vectors encoding IFITM proteins, 18 µg of pQCXIP-based vectors encoding IFITM proteins or *cat*, 9 µg MLV-gag-pol plasmid and 9 µg VSV-G expression plasmid were calcium phosphate transfected into 293T cells seeded into T75 flasks. The culture medium was exchanged at 6 h post transfection and supernatants were harvested at 2 days post transfection. Supernatants were passed through 0.45-µm-pore-size filters and stored at −80°C. To produce MLV reporter particles pseudotyped with different viral glycoproteins, 293T cells seeded at 60% confluence were transfected in T25 flasks using calcium phosphate precipitation with 3 µg plasmid encoding MLV gag pol, 6 µg plasmid encoding a luciferase-bearing MLV vector and 3 µg plasmid encoding for the viral entry proteins (WSN HA/NA, LASV-GPC, envelope protein from MLV or glycoprotein of VSV. At 48 hours after transfection, supernatants were harvested and filtered through a 0.45 µm-pore-size-filter.

### Retroviral Transduction

To measure inhibition of HA-driven viral entry by U18666A, 293T cells were seeded in 96-well plates at a density of 2×10^4^ cells per well. One hour before infection with WSN HA/NA-pseudotyped MLV-particles, the cells were preincubated with 50 µl medium containing two times the final inhibitor concentration. After one hour, 50 µl of pseudotypes were added and the cells were incubated for 8 hours at 37°C. Thereafter, the supernatants were removed and 150 µl of fresh medium without inhibitor was added to the cells. Firefly-luciferase activity in cell lysates was analyzed at 72 h after infection.

For the generation of IFITM expressing cells, cells (293T cells if not stated otherwise) were seeded in a 96-well plate at a density of 10^4^ cells per well. The adherent cells were transduced with the IFITM encoding MLV-based vectors (see above) by centrifugation at 4000×g for 30 min at room temperature. The transduced cells were co-incubated with the infection medium for 48 hours before the supernatant was replaced by 50 µl of fresh medium. Subsequently, the cells were inoculated with 50 µl of infectivity-normalized pseudotypes and incubated for 8 hours at 37°C. Thereafter, the supernatants were removed and 150 µl of fresh culture medium were added per well. Firefly luciferase activity was analyzed at 72 h after transduction.
